# IRES-Mediated Translation: Expanding the Toolkits of RNA Therapy

**DOI:** 10.3390/ijms262110542

**Published:** 2025-10-30

**Authors:** Xiang Gao, Zhenfang Wu

**Affiliations:** 1 Key Laboratory of Systems Health Science of Zhejiang Province, School of Life Science, Hangzhou Institute for Advanced Study, University of Chinese Academy of Sciences, Hangzhou 310024, China; 2 State Key Laboratory of RNA Innovation, Science and Engineering, New Cornerstone Science Laboratory, CAS Center for Excellence in Molecular Cell Science, Shanghai Institute of Biochemistry and Cell Biology, University of Chinese Academy of Sciences, Shanghai 200031, China

**Keywords:** IRES, translation, cap-independent translation, RNA therapeutic, ITAF, circRNA, cell-specific expression, ASO

## Abstract

RNA therapy appears to be a promising strategy to treat various diseases. In recent years, mRNA vaccines have shown notable efficacy in preclinical studies for cancer vaccines, autoimmune disease, and pandemic intervention. Internal ribosome entry sites (IRESs) are structured RNA elements to initiate translation independent of 5-cap recognition of mRNA, particularly show efficient activity under disease stress that causes global canonical translation repression. Studies on distinct structural properties and interaction with translational factors have revealed the mechanisms and regulation of IRES-mediated translation. This allowed the application of IRES for cap-independent translation and dynamic modulation of protein expression in response to cell signals. In this review, we discuss the current platforms and emerging strategies for employing IRES-mediated translation towards novel RNA therapeutics.

## 1. Introduction

Over the past decades, technological innovation in RNA synthesis, modification, and delivery has enabled in vitro transcribed messenger RNA (mRNA) as a promising alternative to conventional vaccine and protein replacement therapy [1,2]. Engineered mRNAs have achieved programmable protein production with high efficiency, design flexibility, and rapid manufacturing compared to many traditional approaches [3], which allowed fast development of vaccines against the COVID-19 pandemic and adaptation to emerging virus variants [4,5,6]. In addition to infectious viruses, mRNA-based therapeutics also show the capability to address a wide range of challenging diseases, including cancer, metabolic and genetic disorders, and cardiovascular and cerebrovascular pathologies [7,8,9]. The translational efficiency of mRNA is a critical parameter for mRNA-based drugs, which directly influences delivery dosage and therapeutic effect. In eukaryotes, the canonical cap-dependent translation initiates with recognizing the 5-m^7^GpppN cap structure of mRNAs by eukaryotic initiation factor 4F (eIF4F), comprised of eIF4E, eIF4G, and eIF4A, to recruit 43S complexes. Subsequently, the 48S complex scans 5′ UTR for the start codon to trigger the joining of the 60S subunit and the formation of an elongational 80S ribosome [10,11]. However, canonical translation is often globally repressed under stress [12,13] or during certain viral infections [14]. This shutdown serves as a host defense mechanism and simultaneously brings a shift from cap-dependent to cap-independent translation to synthesize essential stress-responsive proteins and survival factors of cells or viruses [15].

Activation of cap-independent translation can be achieved by several mechanisms, such as m^6^A modification [16,17], cap-independent translation enhancer (CITE) [18], and the most characterized element, the internal ribosomal entry site (IRES) [19,20,21,22,23]. IRESs are structured RNA elements that recruit the ribosome independently of the 5′ cap by forming RNA–RNA and RNA–protein interactions with ribosomal subunits, bypassing the requirement of one or more eIFs, depending on the IRES classes [24,25,26]. Viral IRES elements are typically classified into four classes based on their secondary or tertiary structure, requirement for eIFs, and IRES-trans-acting factors (ITAFs) [10,27]. The class I IRESs (e.g., Polivirus, PV) require most eIFs except for eIF4E, to recruit the 43S preinitiation complex with eIF4G and eIF4A [19,28,29]. Class II IRESs (e.g., Encephalomyocarditis virus, EMCV) require similar eIF requirements, but initiate translation directly at the start codon without scanning, thereby circumventing the need for eIF1 [20,30,31,32]. Importantly, in addition to canonical factors, class I and class II IRESs rely on ITAFs, such as pyrimidine-region-binding protein (PTB), ITAF45, and poly-C-binding protein-2 (PCBP2), to stabilize their structures and enhance ribosome recruitment [28,33,34]. Class III IRESs (e.g., Hepatitis C Virus, HCV) fold to a compact tertiary structure that directly engages 40S and eIF3, bypassing most other canonical factors [32,35,36]. Class IV IRESs (e.g., Cricket Paralysis Virus, CrPV) can bind with the ribosome in an elongation-competent state without canonical eIFs, using RNA mimicry of tRNA–mRNA interactions to position the ribosome directly [37,38,39]. Translation initiation, bypassing the requirement of specific eIFs, provides an elegant way for the virus IRES to take advantage of the major cellular antiviral response mechanism. In fact, IRESs from HCV and CrPV support robust synthesis of viral proteins during eIF2α phosphorylation that reduces the availability of the eIF2–GTP–Met-tRNA_i_ ternary complex and thereby suppresses canonical cap-dependent initiation [40,41,42]. In mammalian cells, about 10% of mRNAs contain IRES elements [43,44]. These IRESs contribute to the translation of proteins essential for cell function and cell stress response, including those involved in apoptosis regulation (XIAP [45], Bcl-2 [46], Apaf-1 [22]), cell cycle (p27 [47], p53 [48], PITSLRE), proliferation (c-myc [49], c-Jun [50]) and hypoxia adaptation (HIF-1α [51], VEGF [52], FGF2 [53]). Compared with viral IRESs, most cellular IRESs are structurally heterogeneous and less compact, relying on ITAFs to facilitate RNA folding and recruit ribosomes [54,55]. Although the exact complement of required eIFs remains poorly defined for many cellular IRESs, in many cases, some canonical eIFs like eIF4E are dispensable [26,54].

Given their capacity to initiate translation independent of the 5′ cap, IRES elements have emerged as compelling therapeutic tools for driving protein synthesis in non-canonical contexts. Significantly, IRESs offer opportunities for circular RNAs (circRNAs) [56,57], which lack the 5′ cap structure and poly A tail [58,59], by enabling sustained protein expression lasting weeks or months in vivo [60,61]. The durable protein translation of circRNAs has demonstrated remarkable therapeutic potential for cancers or viral infections by getting rid of the limitation of the rapid degradation of conventional linear mRNA [62,63,64,65]. In addition, the capability of IRESs to recruit ribosomes depends on their well-organized RNA structure [66], small molecules or antisense oligonucleotides targeting viral IRESs have been proposed as novel antiviral strategies to specifically impair pathogen infection without perturbing host translation [67].

In this review, we discuss the potential applications of IRES elements as therapeutic tools and targets and explore how to modulate IRES translation to realize conditional protein expression with RNA-based modalities in biomedical research and therapeutics.

## 2. IRES-Based Platforms for Therapeutic Protein Expression

**Figure 1:**
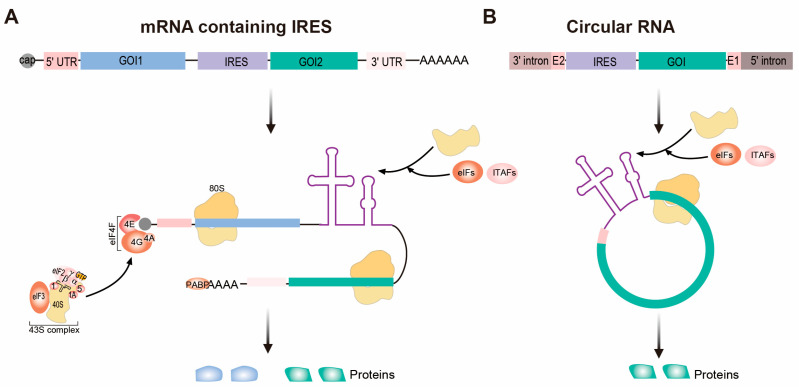
IRES-based platforms for therapeutic protein expression. (**A**) Expression of multiple genes from one linear mRNA transcript is achieved by incorporating an internal ribosome entry site (IRES) element. For upstream canonical translation initiation, the eIF4F complex, comprising eIF4E, eIF4G, and eIF4A, binds the 5′ cap of mRNA and facilitates the recruitment of the 43S complex, which consists of 40S ribosomal subunit, eIF3, GTP-bound eIF2 ternary complex, and the initiator tRNA (eIF2–GTP–Met-tRNA_i_), and eIF1, eIF1A, and eIF5. PABP dynamically engages the 3′ poly(A) tail of many mRNAs and interacts with eIF4G to promote ribosome recruitment. Then the 48S complex scans the 5′ UTR to locate the start codon in an ATP-dependent manner. This triggers the release of initiation factors and subsequent joining of the 60S large ribosomal subunit, thereby generating the elongational 80S ribosome. For internal noncanonical translation, the IRES element recruits the ribosomal subunits through specialized RNA structures either directly or through interaction of eIFs and ITAFs, independent of 5′ cap and 3′ poly(A) tail. (**B**) CircRNAs produced via an autocatalytic group I intron mechanism can be engineered for protein expression using IRES elements. GOI, gene of interest. UTR, untranslated region. eIF, eukaryotic initiation factor. PABP, poly(A)-binding protein. ITAF, IRES *trans*-acting factor.

Combined gene therapy has been displayed as an attractive approach to improve disease treatment. Multicistronic constructs can utilize IRESs positioned between open reading frames (ORFs) to drive translation of a downstream gene from the same RNA transcript, ensuring coordinate expression of multiple proteins [68,69,70] (Figure 1A). Importantly, the independent translation pattern of multiple ORFs avoids the limitation of self-cleaving 2A peptides, which may lead to stoichiometric imbalances and fusion protein artifacts that compromise therapeutic function [71,72]. In fact, IRES-based multicistronic vectors have demonstrated therapeutic benefits across diverse diseases, such as cancer [73,74,75,76], cardiovascular diseases [77,78] and degenerative diseases [79]. In hindlimb ischemia models, bicistronic vector delivery of fibroblast growth factor 2 (FGF2) and cellular communication network factor 1 (Cyr61) with FGF1 IRES stimulated synergistic angiogenesis with significantly lower doses of the angiogenic factors, and avoided systemic undesirable effects on accelerating B16 melanoma growth caused by robust expression from a monocistronic vector [77]. Although vascular endothelial growth factor (VEGF) is a critical regulator of angiogenesis [80], robust overexpression of VEGF has been associated with deleterious effects like vessel instability and may be insufficient to drive durable and functional vessel formation in vivo [81,82,83]. This highlights the requirement of simultaneous expression of various growth factors. Consistently, IRES-mediated co-expression of VEGF and bone morphogenetic protein 7 (BMP) in rabbit mesenchymal stem cells stimulated simultaneous bone formation and vascular regeneration [78]. Similarly, combined protein expression of VEGF and FGF-4 with IRES in mice has demonstrated improved vessel recovery and reduced necrotic events in the acute limb ischemia model [84].

Generally, protein expression from a downstream ORF using an IRES in a multicistronic vector is often lower than that of the upstream ORF via the cap-dependent mechanism. This is partly because typical IRESs like Encephalomyocarditis virus (EMCV) IRES, exhibit shorter and rarer bursts of translation initiation than the cap-dependent process in normal condition [85]. By incorporating IRES elements with enhanced translational efficiency and strategically positioning them within the vector, translation of multiple ORFs can be balanced to achieve desired outcomes [86,87,88]. For instance, retroviral bicistronic vectors delivering Interleukin-12 (IL-12) subunits with different IRESs have achieved efficient protein output approaching that of a single-chain IL-12 construct, particularly for the high efficiency of poliovirus IRES in melanoma cells [89]. These studies have shown that IRES-based multicistronic vectors represent a practical platform in combined gene therapy, enabling balanced co-expression of multiple genes from a single transcript. However, selection of appropriate IRES elements for different genes, constraints of IRES length on vector packaging capacity, and translation efficiency in specific cell types, still require further investigation to expand the scope of multicistronic vectors in combined gene therapy of various diseases.

In addition to linear mRNAs, circular RNAs (circRNAs) have emerged as a novel class of RNA therapeutics due to their enhanced stability and low immunogenicity [60,61,90]. Although generally having low efficiency under normal conditions, evidence has shown that a subset of endogenous circRNAs can translate proteins [91,92,93,94,95]. Certain circRNAs harbor structured RNA elements complementary to regions of the 18S rRNA, which may facilitate direct ribosomal recruitment in a cap-independent manner [91]. In addition, m^6^A modification has been identified as a key regulatory mark that enables internal translation initiation of circRNAs [93,94,95]. Yang et al. demonstrated that m^6^A residues within circRNAs can recruit the initiation factor eIF4G2 and the m^6^A reader protein YTHDF3 to promote ribosome assembly [93]. This capability has led to the speculation that synthetic circRNAs can be engineered for sustained protein production, due to their exonuclease resistance and reduced innate immunogenicity without nucleotide modifications [60,61]. However, lacking a 5′ cap, circRNAs require internal translation initiation elements to initiate translation [61,96,97] (Figure 1B). Proof-of-concept studies have shown synthetic circRNAs incorporating efficient IRES elements produced via an autocatalytic group I intron mechanism, can be engineered for prolonged protein production in vivo than linear mRNAs upon nanoformulated delivery [60,61]. In drug development, a circRNA vaccine expressing the trimeric receptor-binding domain (RBD) of the SARS-CoV-2 spike protein by CVB3 IRES was designed to provide more effective and broad-spectrum protection [64]. This circRNA^RBD^ vaccine generated significantly higher and more durable antigen production compared to 1-methyl-pseudouridine (1mΨ) modified mRNA vaccines in both mice and rhesus macaques [64]. In addition, the circRNA vaccine platform can also be applied for enhanced cancer immunotherapy. A circRNA vaccine including HRV-B3 IRES and encoding ovalbumin (circOVA) elicited strong CD8^+^ T-cell responses and induced systemic antitumor effects and abscopal tumor regression in murine melanoma models [65]. Compared to linear mRNA controls, the circRNA vaccine expressing hepatocellular carcinoma-specific tumor neoantigens using Coxsackievirus B3 (CVB3) IRES displayed robust in vitro dendritic cell activation and significant tumor growth inhibition and enhanced survival in a mouse model [98]. Similarly, the circRNA vaccine incorporating Enterovirus-A (EV-A) IRES to express multiple tumor neoantigens, elicited high levels of neoantigen-specific CD8^+^ T cell responses, dendritic cell activation, and durable tumor regression in murine models of melanoma and hepatocellular carcinoma, while the control groups receiving traditional linear mRNA vaccines or non-optimized circRNAs showed significantly weaker immunity and inferior tumor control [99]. Collectively, these studies show that IRES-based circRNA platforms enable durable antigen expression, precise immune activation, and superior safety profiles for cancer immunotherapy and infectious disease interventions.

Although IRES-based circRNA therapeutics have demonstrated encouraging effects, the rational and rapid design of circRNAs incorporating efficient IRES elements remains challenging. For linear mRNAs, the translation efficiency can be largely enhanced through codon adaptation index (CAI) and secondary structure optimization [100,101,102,103]. In contrast to linear mRNAs, IRES activity is also crucial for circRNAs to translate abundant proteins [104,105]. By screening efficient IRES elements and incorporating eIF4G-recruiting RNA aptamers, engineered UTRs, and m6A modifications, Chen et al. enhanced NanoLuc reporter expression from circRNAs to several hundred-fold [106]. Introducing nucleotide mutations and deletions to streamline RNA secondary structure, and optimizing spacer sequences and microRNA recognition elements of screened EV-A IRES, significantly enhanced antigen expression in vivo, compared to unoptimized circular and linear mRNA vaccines [99]. However, commonly used IRESs, such as IRESs of EMCV and CVB3, are approximately 500–750 nucleotides (nt) in length, which compromise the circularization efficiency of long circRNAs. A synthetic oligonucleotide library comprising thousands of designed sequences about 200 nt from human and viral genomes, including putative regulatory regions [44] was screened using an oligo-split-eGFP-circRNA reporter, which showed a structured RNA element (SuRE) and complementarity to 18S rRNA, which can facilitate cap-independent translation on circRNA [91]. Further engineering of short IRES-like elements is still desired to reach comparable efficiency with full-length viral IRESs. In addition to IRES engineering, whether IRES adopts well-organized structure also influences ribosome recruitment and processivity of circRNAs [107]. Although circRNAs lack traditional 5′ and 3′ UTRs, the sequences immediately flanking the IRES and ORF can modulate local RNA folding and accessibility, thus appropriate sequence optimization and additional spacers may facilitate IRES function by reducing unintended base pairing between IRES and other regions [108]. An emerging strategy involves artificial intelligence and deep-learning methods to accelerate IRES-based circRNA vaccine design [109,110]. The AI-driven pipeline can employ deep-learning models to optimize the ORF sequences, balancing codon usage and RNA secondary structures, delivery system, circularization efficiency, and IRES activity, to achieve a desired output superior to traditional computational approaches [110]. Given that current algorithms for recapitulation and analyses of conformation of translatable circular RNA containing IRES still remain challenging, further progress combining AI models with traditional mechanistic screens is required toward advancing circRNA vaccine development.

## 3. Modulation of IRES-Mediated Translation for Conditional Expression

A defining advantage of IRES-mediated translation lies in its capability of enabling protein synthesis under circumstances where cap-dependent initiation falters [14,111]. More broadly, various cellular stresses have been shown to trigger IRES activity in mRNA transcripts governing survival and adaptation [15,112], which is mediated by IRES *trans*-acting factors (ITAFs) that sense environmental stimulation and modulate translation [54,113]. Benefiting from this modality, IRES-mediated translation has shown attractive potential for conditional expression of therapeutic proteins.

### 3.1. Predominant Translation of IRES Under Stress Conditions

**Figure 2:**
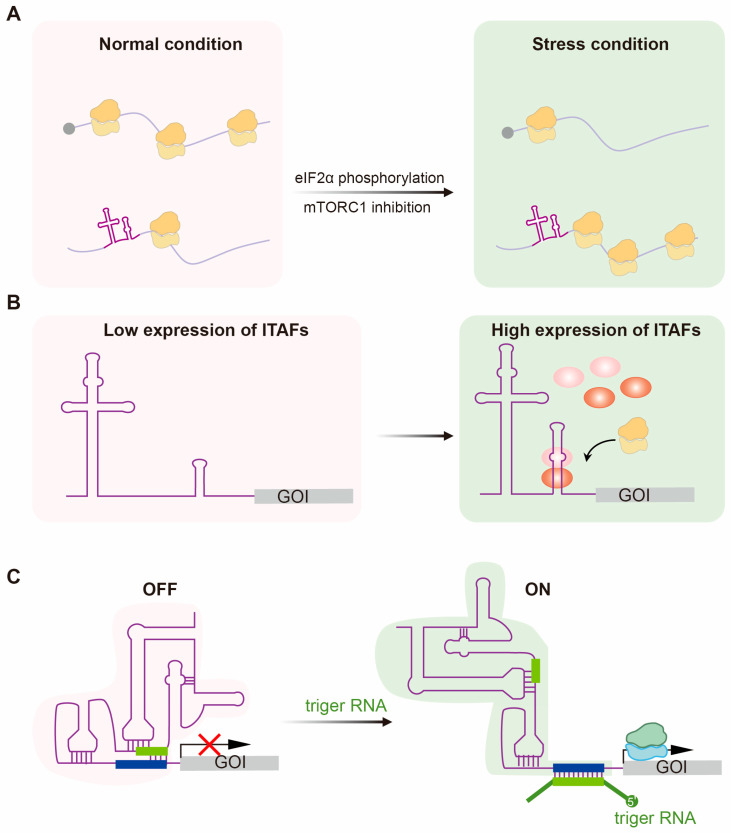
Modulation of IRES-mediated translation for conditional expression. (**A**) Cells adapt to stress by reducing global canonical translation while maintaining the selective production of survival and stress-responsive factors through IRES-mediated mechanisms. This translational shift is primarily driven by phosphorylation of eIF2α or by mTORC1 inactivation. (**B**) Cell-specific expression can be realized by ITAF binding that regulates IRES-mediated translation. In cells with low levels of required ITAFs, the given IRES remains largely inactive, limiting ribosome recruitment and protein synthesis. While in cells that express abundant ITAFs, the same IRES adopts an active conformation and binds these ITAFs to modulate internal translation initiation. (**C**) Engineered IRES switch responses to trigger RNAs, leading to conformational and initiation translation. Left, the inserted sequences lead to an inhibitory conformation that blocks ribosome access and prevents downstream translation. Right, hybridization of an exogenous or endogenous trigger RNA remodels the IRES into a functional structure that can recruit ribosomes and drive translation initiation. GOI, gene of interest. ITAF, IRES *trans*-acting factor.

Eukaryotic cells utilize non-canonical translation mechanisms to ensure the synthesis of critical proteins when global cap-dependent initiation is compromised under stress conditions (Figure 2A). Stresses such as nutrient deprivation, viral infection, oxidative damage, and endoplasmic reticulum (ER) stress cause phosphorylation of eukaryotic initiation factor-2α (eIF2α) or inhibition of mammalian target of rapamycin (mTOR) signaling [14,114,115]. In the integrated stress response (ISR), specialized kinases sense distinct stresses and phosphorylate eIF2α, which sequesters eIF2B required to regenerate eIF2–GTP, thereby reducing the availability of eIF2–GTP–Met-tRNA_i_ [114]. As a result, global translation initiation declines, while a subset of mRNAs harboring IRESs or upstream open reading frames (uORFs) can bypass this repression to ensure selective translation of stress-adaptive proteins [116]. Meanwhile, stress conditions that inhibit the mTOR pathway reduce phosphorylation of 4E–binding proteins (4E–BPs), which associate with eIF4E and inhibit its binding to the 5′ cap and further recruitment of eIF4G [115]. This impairs assembly of the eIF4F complex and recruitment of ribosomes to the 5′ cap of mRNAs, while IRES-mediated translation can still proceed without eIF4E. For example, the 5′ UTR of hypoxia-inducible factor 1–Alpha (HIF-1α) drives translation of the downstream coding sequence even under hypoxic conditions, enabling selective synthesis of HIF-1α protein under low-oxygen stress, a critical adaptive response that activates angiogenic and metabolic gene networks when broad cap-dependent protein synthesis declines [51,117]. In addition, investigations have revealed that apoptotic protease activating factor-1 (Apaf-1) mRNA contains a functional IRES that drives cap-independent translation of this apoptotic initiator [22], which is stimulated by the binding of polypyrimidine tract-binding protein (PTBP1) and upstream of N-ras (unr) [118]. Notably, UV irradiation impairs cap-dependent initiation while enhancing Apaf-1 IRES-mediated translation, thereby promoting caspase activation and apoptotic progression [119]. Similarly, cap-dependent translation is often inhibited in virus-infected cells, either as a host antiviral strategy or due to viral sequestration of translational factors [14,120,121]. Under this situation, many viral IRES elements utilize fewer eIF requirements or alternative factors to ensure their own protein synthesis [27,122]. For instance, the N-terminal eIF4E–binding domain of eIF4G can be cleaved by the protease 2Apro of Poliovirus (PV) or 3Cpro of EMCV, while their IRES elements utilize the remaining eIF4G fragment to recruit 43S complex for translation initiation, bypassing eIF4E [123]. In addition, HCV IRES is a highly structured RNA element and can directly bind to the eIF3 and 40S ribosomal subunit [124]. The most elegant intergenic IGR IRES, such as CrPV IRES, can assemble 80S ribosomes entirely without canonical initiation factors to initiate translation [37]. In these ways, viral IRESs have been proposed to render active translation under global repression of host translation. In fact, studies have shown that HCV IRES-driven translation remains active even when eIF2α is phosphorylated or eIF4A is inhibited, whereas host cap-dependent translation is blocked [66,125]. Consistently, Single-molecule dissection of translation in live cells has revealed that stress conditions cause a functional shift from cap-dependent translation to IRES-mediated translation [85], confirming the adaptation of IRES elements in stress response. This suggests that IRES-mediated translation is a feasible strategy for producing therapeutic proteins under stress conditions when cap-dependent translation is repressed. Such expression can be achieved using either viral IRES elements or cellular IRES derived from stress-responsive genes.

In addition, certain stress-induced ITAFs become abundant in diseases and can enhance or repress IRES activity [126]. Based on the high expression of eIF4G2 and PTBP1 in cancer tissues, Feng and colleagues developed a circular RNA therapeutic that contains Human rhinovirus type 2 (HRV2) IRES to achieve selective translation in cancer cells overexpressing these factors [127]. By fusing a gasdermin D mutant to a mitochondrial-targeting peptide, they harnessed mitochondrial inner membrane cardiolipin toxicity to trigger mitophagy-mediated tumor cell death, which markedly suppressed tumor growth in xenograft models of EIF4G2+/PTBP1+ adenocarcinomas [127]. These results indicate the feasibility of precision gene therapy with IRES-mediated translation under pathological stress conditions.

### 3.2. Cell-Specific Translation of IRESs

While many IRES elements function broadly, some cellular and viral IRESs exhibit cell-type or tissue-specific activity, which can be applied for targeted gene therapy (Figure 2B). A classic example is the fibroblast growth factor (FGF) family. The IRES of the FGF-2 mRNA is inefficient in most tissues except the brain and testis; similarly, the FGF-1 IRES is specifically active in skeletal muscle [128,129]. The FGF-1 IRES has been successfully employed in a bicistronic AAV vector, in which two reporter genes were separated by the 341-nt human FGF-1 IRES, to achieve robust and balanced co-expression of therapeutic genes in skeletal muscle [130]. Following intramuscular injection in mice, both transgenes were expressed for at least 120 days, with downstream gene expression via the FGF-1 IRES being approximately 10-fold higher than that using the EMCV IRES in the same context [130].

Some viral IRESs also show tissue preference, mainly due to the expression of specific ITAFs or RNA-binding proteins [131,132,133]. IRES within the Human immunodeficiency virus (HIV-1) gag transcript drives much higher translation in T cells than HeLa and HEK293 cells, modeling the natural target cells for HIV-1 infection [134]. Similarly, the IRESs of Hepatitis A virus (HAV) and HCV are more active in hepatocytes compared to other cell types, suggesting a preferred context for translation in liver-specific cells [135,136]. In contrast, IRES of Theiler’s murine encephalomyelitis virus (TMEV) directs efficient translation in peripheral cells but is attenuated in neurons due to the absence of canonical PTBP1 [131,133]. Furthermore, the HRV2 IRES in a chimeric virus PV-RIPO is suppressed in neurons by the double-stranded RNA-binding protein DRBP76, yet remains active in glioma and other tumor cell lines, leading to strong anti-tumor potency with reduced neuropathogenicity [137].

Importantly, targeted RNA drug therapeutics encompass diverse, complementary approaches that jointly enhance cell-specific protein expression. Molecular recognition of delivery systems can be achieved by various strategies, such as formulating LNPs through lipid chemical structures and composition adjustments [138], antibody or peptide conjugated LNPs for cancer and immune cells transfection [139,140,141], SORT LNP platform with altered ionizable lipids to confer organ-specific tropism [142,143], and GalNAc-siRNA conjugates for hepatocyte targeting [144]. However, these approaches involve trade-offs in designing novel ligands for each cell type and in manufacturing complexity. Exosome vesicles offer biological compatibility with low immunogenicity, but face challenges in scale and batch variability [145]. In addition to targeted delivery systems, smartly designed vectors like those integrating miRNA-responsive UTRs, also support post-delivery cell specificity [146]. The cell-specific IRES translation alternatively provides an intrinsic mechanism for selective translational control post-delivery. Furthermore, combining these approaches may enhance multilayered specificity and maximize therapeutic efficacy and safety across diverse RNA-based treatments. The challenge in this specificity lies in elucidating the interactions among IRES elements and required ITAFs, and in engineering IRES sequences with tightly restricted activity tailored to defined cell types.

### 3.3. Responsive IRES Switches

Beyond natural cell specificity, IRES elements, especially for structurally well-organized viral IRESs, can be engineered as RNA-responsive switches for conditional translation. In mammalian cells, translational switches are designed by embedding sensing modules into mRNA UTRs to reconfigure ribosome access or mRNA stability [147]. Aptazyme-based OFF-switches couple small-molecule aptamers (e.g., tetracycline, theophylline, guanine) to self-cleaving ribozymes, while tandem aptamer–ribozyme ON-switches stabilize the ribozyme in a non-cleaving conformation upon ligand binding [148,149]. Protein-responsive switches use orthogonal RNA-binding proteins and cognate aptamers in the 5′-UTR to sterically block translation initiation [150]. miRNA-responsive on-switches rely on miRNA-induced cleavage to release polyadenosine tail [151,152] or kozak sequence [153]. Especially, Zhao et al. developed *eToehold* switches by inserting trigger RNA (trRNA) complementary sequences into viral IRESs that structurally modulate translation initiation [154] (Figure 2C). In the default state, the engineered IRES is ‘locked’ by these inserts and cannot recruit the ribosome, while a specific trigger RNA base-pairs with the insert sequences and unfolds the IRES structure to restore its function [154]. Despite miRNAs, the eToehold switches can also sense long RNAs, including intracellular transcripts indicative of cell state or cell type, or exogenous viral RNAs [154]. Furthermore, smartly designed secondary structures with miRNA-binding sites (miRBSs) inserted into the HCV IRES have increased the specificity and efficiency of programmable translational switches [155,156]. Surprisingly, the IRES switches are extremely suitable for circRNA therapy, when traditional control strategies like cleavage-based release of UTR or ribosome loading site are unavailable. CircRNAs containing IRES of HCV or Classical swine fever virus (CSFV) have realized targeting expression in HEK293 and Huh7 cells by incorporating corresponding miRBSs [155]. This strategy was further extended by replacing miRBSs with protein-binding sites and using other IRES elements [156]. Together, the development of IRES switches that integrate regulatory RNA or protein binding sites offers a new approach for cell-specific gene expression. This not only inspires desired RNA therapeutics in a manner responsive to the physiological state of target cells while minimizing off-target effects in non-relevant tissues, but also addresses the low cap-dependent translation efficiency observed in certain pathological conditions. To dissect the RNA structure dynamics of different IRES elements and identify disease-associated RNA transcripts and proteins would further improve the performance and specificity of IRES switches.

## 4. Therapeutically Targeting IRES-Dependent Translation

**Figure 3:**
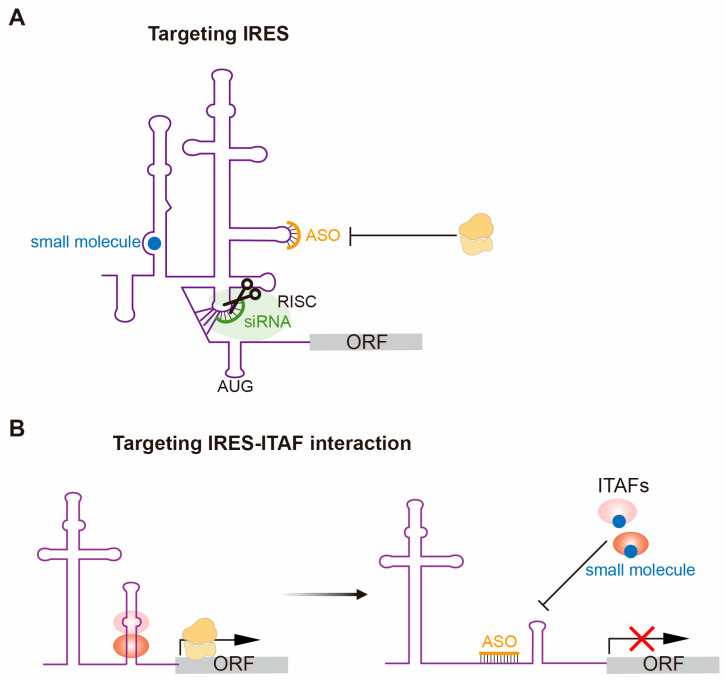
Strategies for therapeutically targeting IRES-dependent translation. (**A**) Multiple approaches can be employed to disrupt IRES structure or prevent IRES interaction with the ribosome to inhibit translation, such as small molecules and ASOs for steric blocks of ribosome assembly, or siRNAs for RNA-induced silencing complex (RISC) mediated RNA degradation. (**B**) Selective blocking of ITAF binding to IRES elements represses ribosome recruitment and cap-independent translation.

Since viral IRESs are generally highly structured, conserved, and essential for viral life cycles [15,27,122], they represent promising therapeutic targets for inhibiting ribosome recruitment. Distinct approaches to target IRES-mediated translation have been reported [67], such as antisense oligonucleotides (ASOs), small interfering RNAs (siRNAs), RNA aptamers, small-molecule inhibitors, or peptides. Especially, the HCV IRES forms a well-organized tertiary structure to recruit the ribosome, and various strategies have been employed for targeting HCV IRES [36,124] (Figure 3A). The HCV IRES contains four domains (I–IV), with domains I and II involved in genome replication, while domains II to IV possess IRES activity by interacting with the ribosome [157]. Antisense oligonucleotides (ASOs) can recruit RNase H to cleave the target RNA or induce steric blocking of ribosomes. Early strategies designed ASOs to hybridize with conserved IRES domains, and those targeting the IIId loop or AUG-containing region showed efficient inhibition effect [158,159,160]. Locked nucleic acids (LNAs) and morpholinos further improved affinity and stability for HCV targeting [161,162]. Although modified antisense oligonucleotides achieved encouraging results in vitro or in mouse models, delivery and cell uptake of such modified oligonucleotides are major limitations of this methodology [160,161,163]. SiRNAs can recruit RNA-induced silencing complex (RISC) to cleave complementary IRES-containing viral RNAs, thereby preventing their translation and replication [164,165,166]. A siRNA targeting the IRES subdomain IIIf delivered via LNP formulation achieved an obvious reduction in IRES-driven reporter expression in mouse liver with negligible type 1 interferon induction [167]. SiRNAs can be rapidly designed and manufactured for strong cleavage, but tissue-specific delivery also remains challenging, and the cleavage efficacy may be lost upon target viral sequence mutation. In addition, small molecule inhibitors targeting the subdomain IIa demonstrated sub-micromolar binding affinities and notable replicon inhibition in cells [168]. High-resolution crystal structures revealed a deep ligand pocket for this domain that locks the RNA into an inactive state, enabling structure-based medicinal chemistry [165]. Moreover, inhibitors targeting other viral IRESs (e.g., EV71 IRES, PV1 IRES) also impeded viral replication, addressing antiviral potential by blocking viral IRES-dependent translation [169,170,171,172]. Small-molecule inhibitors are currently preferred for drug therapies, benefiting from their permeability, chemical stability, and screening scalability, while their toxicity requires careful evaluation to exclude potential off-target effects [173].

Dysregulated cellular IRES-mediated translation is associated with numerous human diseases [126,174]. By contrast to viral IRESs, human cellular IRES elements rarely share common sequences or structural homology with viral IRESs. Their activity is mostly controlled by eIFs and specific ITAFs, which introduce conformational change or interact with other translational factors to regulate ribosome binding [54,113]. PTBP1 is a classical ITAF that facilitates translation initiation of picornaviruses by modulating eIF4G binding [175,176]. In addition to viral IRESs, it also contributes to the activity of many cellular IRESs, such as p53 IRES, upon DNA damage [177], cyclin B1 IRES [178] and c-myc IRES [179] in cancer, and HIF-1α IRES in hypoxia [51]. Heterogeneous nuclear ribonucleoprotein A1 (hnRNPA1) shares the same pattern of translocating from the nucleus to the cytoplasm with PTBP1 to negatively or positively regulate the activity of several IRES [180,181,182,183]. Given the roles of ITAFs in IRES-mediated translation in physiological processes, targeting the IRES-ITAF interaction provides a new approach to therapeutically regulate specific gene expression (Figure 3A). hnRNP A1 can bind directly to the IRES regions of cyclin D1 and c-myc to regulate their translational efficiency in response to Akt signaling [184]. Following this, an already FDA-approved drug, riluzole for amyotrophic lateral sclerosis, was identified as an hnRNP A1 inhibitor. This drug has decreased the activity of cyclin D1 and c-myc IRES and showed synergistic anti-GBM efficacy by combining with mTOR inhibitors [185]. Similarly, antisense oligonucleotides targeting ITAF binding sites are expected to unlock more promises of IRES-based therapeutics in the near future.

Therapeutic targeting of IRES-dependent translation represents an encouraging strategy for modulating pathogenic gene expressions in disease contexts [67,174,186]. Potential approaches can be achieved by directly disrupting IRES RNA structures or by blocking ITAFs and ribosome recruitment to inhibit translation of pathogenic transcripts. Although small-molecule drugs remain challenging to achieve sufficient potency and selectivity in vivo while avoiding off-target effects on cellular IRESs, viral IRES targeting is appealing due to low mutation rates in the compact IRES structure [187]. Oligonucleotides represent an attractive modality to directly target IRESs or disrupt IRES-ITAF interaction [188], benefiting from their programmability and increasing success in RNA therapeutics. Further work is warranted to better understand IRES structures, associated ITAFs, and cell-specific regulatory networks. It is worthwhile noting that ITAFs can also function independently of IRES-mediated translation or regulate the activity of multiple IRESs. Thus, repression of specific ITAFs may cause unsuspected side effects in normal cells. Strict safety assessment is required to ensure post-transcriptionally fine-tune IRES-mediated translation under pathological states for precision medicine applications.

## 5. Discussion

RNA-based therapies have demonstrated promising effects across diverse diseases, yet challenges remain in optimizing translation efficiency, durability, and cell specificity to realize their potential fully. IRES-mediated translation provides a powerful alternative to expand the scope of current RNA platforms, particularly under disease-relevant pathological states where global translation is impaired, such as hypoxia, endoplasmic reticulum stress, or viral infection [14,111]. In this context, IRES elements can sustain or reprogram protein synthesis to ensure the expression of stress-responsive or therapeutic factors when canonical initiation is compromised.

The application of IRES elements has expanded beyond basic mechanistic studies into practical RNA therapeutic platforms. In linear mRNA systems, IRESs enable the balanced, synergistic co-expression of multiple therapeutic proteins from one mRNA transcript. More importantly, IRESs have inspired the application of circular RNA platforms, which lack the 5′ cap and poly(A) tail but rely on IRES-driven translation for prolonged protein expression. Recent studies have shown that circRNA vaccines incorporating optimized viral or synthetic IRESs produce durable immune responses and enhanced antigen production compared with conventional linear mRNA vaccines [64,98,99,106,189]. Although circRNA vaccines are still in the preliminary stage, future engineering of IRESs to improve translation activity while shortening the length holds promise for driving the evolution of circular RNA platforms. Furthermore, regulation of IRES activity by translational factors or RNA-binding proteins in different contexts offers opportunities for dynamic protein translation in stress-responsive or cell-specific patterns, which is further highlighted by engineered IRES switches in response to various intracellular signals. Integrating these designs of IRES-based translation with advances in delivery systems will accelerate the development of programmable RNA therapeutics.

Despite these advances, several fundamental questions remain unresolved to facilitate IRES-based RNA therapeutics. These include how IRES activity exhibits marked variability across cell types, the molecular basis for IRES structure contributing to ribosome recruitment, and the regulatory network of IRES-associated ITAFs. Systematic dissection of activity of different IRES elements, and AI-based IRES engineering are desired for rational vaccine design and precise modulation of translation.

It is noteworthy that application of cellular IRESs should be careful because the authenticity of many cellular IRESs remains controversial. Most cellular IRESs have been identified using bicistronic reporter assays, which are inherently prone to false-positive artifacts resulting from cryptic promoter activity, alternative splicing, or aberrant RNA processing [26,190]. These sequences function instead as cap-independent translation enhancers (CITE), which promote cap-independent initiation of mRNAs scanned from the 5′ end by recruiting initiation factors [191], rather than acting as classical internal ribosome entry sites. Of note, the circRNA platform is very suitable for evaluating the performance of IRES sequences.

Finally, we optimistically foresee that more exciting IRES-based RNA therapeutics will be developed to benefit biomedical research.

## Data Availability

No new data were created or analyzed in this study.
